# Effect of ambient temperature and other environmental factors on stroke emergency department visits in Beijing: A distributed lag non-linear model

**DOI:** 10.3389/fpubh.2022.1034534

**Published:** 2022-11-16

**Authors:** Jinhua Zhao, Yongming Zhang, Ying Ni, Junyu He, Jianping Wang, Xuan Li, Yuming Guo, Changping Li, Wenyi Zhang, Zhuang Cui

**Affiliations:** ^1^Department of Epidemiology and Biostatistics, School of Public Health, Tianjin Medical University, Tianjin, China; ^2^Department of Pulmonary and Critical Care Medicine, Center of Respiratory Medicine, China-Japan Friendship Hospital, National Clinical Research Center for Respiratory Diseases, Beijing, China; ^3^Ocean College, Zhejiang University, Zhoushan, China; ^4^Ocean Academy, Zhejiang University, Zhoushan, China; ^5^Department of Epidemiology and Preventive Medicine, School of Public Health and Preventive Medicine, Monash University, Melbourne, VIC, Australia; ^6^Chinese PLA Center for Disease Control and Prevention, Beijing, China

**Keywords:** stroke, ambient temperature, environmental factors, generalized additive model, distributed lag non-linear model

## Abstract

**Background:**

Most studies have focused on the relationship between ambient temperature and stroke mortality, but studies on the relationship between ambient temperature and stroke occurrence are still limited and inconsistent.

**Objective:**

This study aimed to analyze the effect of ambient temperature and other environmental factors on emergency stroke visits in Beijing.

**Methods:**

Our study utilized stroke visit data from the Beijing Red Cross Emergency Medical Center during 2017–2018, and applied a generalized additive model (GAM) as well as a distributed lag non-linear model (DLNM), respectively, regarding the direct, lagged, and cumulative effects of ambient temperature alone and with correction for other environmental factors on stroke occurrence.

**Results:**

With a total of 26,984 emergency stroke patients in 2017–2018, both cold and hot effects were observed and weakened after correction for other environmental factors. Compared to the reference temperature, in the multi-factor model, extreme cold (−10°C) reached a maximum relative risk (RR) of 1.20 [95% Confidence Interval (CI): 1.09, 1.32] at lag 14 days, and extreme hot (30°C) had a maximum RR of 1.07 (95% CI: 1.04, 1.11) at lag 6 days. The cumulative effect of extreme cold reached a maximum of 2.02 (95% CI: 1.11, 3.67) at lag 0–14 days, whereas the cumulative effect of extreme hot temperature is greatest at lag 0–10 days, but no statistically significant effect was found. In addition, ischemic stroke patients, the elderly, and males were more susceptible to the effects of cold temperature.

**Conclusions:**

There is a non-linear relationship between ambient temperature and stroke occurrence, with cold temperature having a greater and longer-lasting impact than hot temperature.

## Introduction

Stroke is brain tissue damage caused by rupture or blockage of cerebral blood vessels, which is characterized by high morbidity, disability, mortality, and recurrence (1), and it is a major public health concern facing people in China and throughout the world nowadays. The development of stroke is caused by a combination of risk factors, including abnormal metabolic factors, behavioral factors, and environmental factors (2). Global warming, heat waves, and cold waves, as well as increased air pollution, have become more frequent since the beginning of the twenty-first century, the effect of environmental factors on the incidence of stroke has become more and more significant (3). According to the findings of the Global Burden of Disease Study 2019 (GBD), the burden of disease due to non-optimal temperature and environmental particulate pollution accounts for up to 37% of disability-adjusted life years due to stroke (4).

There has been considerable focus on the association between ambient temperature and stroke death (5–7), but in contrast, the risk of morbidity can describe broader health outcomes and medical costs (8). However, the epidemiological evidence that is now available is inconsistent, and the association between ambient temperature and the incidence of stroke has not been sufficiently studied. A comprehensive study has shown an increased risk of cardiovascular hospitalization under cold temperatures and prolonged exposure to very high temperatures (9). Another study found that whereas hot temperatures were protective against stroke incidence, cold temperatures were a risk factor (10). In addition, other studies have shown no significant correlation between stroke admission and ambient temperature (11, 12). Therefore, the relationship between ambient temperature and the incidence of stroke needs to be further clarified.

The results of a large time-series study showed that the total disease burden caused by non-optimal temperatures was more pronounced in the temperate monsoon climate zone of China, yet Beijing, which has a typical temperate monsoon climate, lacks studies on the correlation between ambient temperature and stroke risk (5, 13). To the best of our knowledge, so far, only one study has focused on the attributable risk of stroke hospitalization in Beijing caused by extreme temperatures from 2013 to 2014 (14). However, given the characteristics of the health care system, compared with stroke hospitalization, emergency admission is a better indicator of population response to fluctuations in environmental factors (15). Our study uses stroke emergency data from the largest emergency center in Beijing from 2017 to 2018, which can more reasonably and accurately reflect the association between ambient temperature and stroke risk. It is a repeatable, high-quality latest assessment based on a large population.

Previous studies have found a correlation between other environmental factors and stroke occurrence (16–20), which may modify the relationship between ambient temperature and stroke (21–23); therefore, in our study, we evaluated the effect of ambient temperature alone and then combined the modified effects of multiple environmental factors to consider their effects on stroke occurrence. Since the effect of ambient temperature on stroke incidence is non-linear and lagged (9, 24, 25), we used a distributed lagged non-linear model to analyze stroke emergency department visit data in Beijing from 2017 to 2018, in addition, we explored the effect of temperature and specific environmental factors on stroke by developing a generalized additive model. This analysis could aid public health policies in Beijing in implementing the proper mitigation and adaptation measures.

## Materials and methods

### Data collection

Daily emergency stroke visits in Beijing from January 2017 to December 2018 were obtained from the Beijing Red Cross Emergency Medical Center. The data included the number of visits, type of stroke diagnosis, gender and age of patients, and stroke subtypes [ischemic stroke (IS) and hemorrhagic stroke (HS)]. The meteorological data and air pollution data for the same period were obtained from the online publication platform of the Chinese National Environmental Monitoring Center (http://106.37.208.233:20035). The meteorological data include daily mean temperature, daily mean relative humidity, daily mean air pressure, mean wind speed, and daily precipitation, and the air pollution data include daily mean levels of *PM*_2.5_, *PM*_10_, sulfur dioxide (*SO*_2_), nitrogen dioxide (*NO*_2_), ozone (*O*_3_), and carbon monoxide (*CO*).

### Statistical analysis

First, the stroke data were cleaned and organized into daily-scale time series data, and descriptive statistics were used to describe patient characteristics, including gender, age group, and stroke subtype. Time series plot was used to assess the periodicity and long-term trends of stroke visits and daily mean temperatures. Second, because daily stroke incidence for the total population is a low probability event that roughly follows a Poisson distribution, the generalized additive model (GAM) was employed to investigate the association between each environmental element and the frequency of stroke visits.

We then carried out a two-stage analysis. In the first step, we developed a univariate distributed lagged non-linear model (DLNM) of daily mean temperature, combined with GAM to analyze the non-linear relationship, lagged effect, and the cumulative effect of mean temperature on stroke incidence. We established a cross-basis matrix for assessing the two-dimensional relationship between different lag days and mean daily temperature (26), using the natural logarithm of the number of daily stroke visits as the dependent variable and the natural cubic spline function as the basis function for the exposure-response association and the lag-response association, setting the degrees of freedom of the exposure dimension to 6 and the degrees of freedom of the lag dimension to 4 based on previous studies and the results of the generalized deficit pool information minimization criterion (27). According to previous studies, the effect of hot temperature on stroke incidence is within a week, and the cold effect lasts longer. In general, the use of shorter lags may not capture the potential harvest effect of hot temperature, in reference to previous studies, so our study set the maximum lag days to 14 days (28–30). After controlling for day-of-week effects, long term and seasonality, the model we depicted is shown below:


(1) $$\begin{array}{r} Y_{t}\sim Poisson\left(\mu_{t}\right)\\ Log\left[E\left(Y_{t}\right)\right]=\alpha +\beta^{*}Temp_{t,l}+\;ns\left(time,12^{*}2\right)\\ +\eta DOW_{t} \end{array}$$

where *Y*_*t*_ is the number of stroke visits on day t, α is the intercept, β and η are the regression coefficients, and *Temp*_*t, l*_ is the temperature cross basis function established in DLNM, taking into account both the non-linear relationship of ambient temperature and the lag effect from lag 0 (the day of exposure) to lag l, where the maximum value of l is 14 days. *ns* is the natural cubic spline curve, and we used 12 degrees of freedom per year to control for long-term and seasonality, with degrees of freedom chosen with reference to previous research and in accordance with the Akaike information criterion(AIC) minimization (5). *DOW*_*t*_ is the day-of-week effect and is included in the model as a categorical variable. In this study, the ambient temperature was classified as extreme cold (1st percentile of temperature), moderate cold (10th percentile of temperature), moderate hot (90th percentile of temperature), and extreme hot (99th percentile of temperature). The above were compared with reference values to calculate the relative risk (RR) [95% confidence interval (CI)] of emergency stroke visits for different ambient temperature exposures. The above cutoff values for ambient temperature were reasonable choices based on previous studies (14, 31, 32), in addition to which we explored the RR of the single-day lag effect for 14 days at these four ambient temperatures, as well as the RR of the cumulative effect at different ambient temperatures with different lag days, specifically lag 0–3 days, lag 0–7 days, lag 0–10 days, and lag 0–14 days.

In the second stage, in order to comprehensively consider the corrective effects of various environmental factors, we further developed a multi-factor model with ambient temperature as the main variable. In order to avoid multi-collinearity among variables, Spearman test was used to explore the correlation among variables, and factors with correlation coefficients >0.6 were not included in the model at the same time (18). Combined with the GAM results, it was finally determined that daily temperature variation (TV), mean relative humidity, *PM*_2.5_, *SO*_2_ were included in the multi-factor model, and the model established was as follows:


(2) $$\begin{array}{r} Log\left[E\left(Y_{t}\right)\right]=\alpha_{1}+\beta_{1}^{*}Temp_{t,l}+\beta_{2}^{*}TV+\;ns\left(RH,3\right)\\ +\beta_{3}^{*}PM2.5+\beta_{4}^{*}SO_{2}\;\\ +\;ns\left(time,12^{*}2\right)+\eta_{1}DOW_{t} \end{array}$$

In equation (2), *α*_1_ is the intercept, *β*_1_, *β*_2_, *β*_3_, *β*_4_ and *η*_1_ are the regression coefficients, and the parameters in *Temp*_*t, l*_ and *time* are set the same as in equation (1). *TV*, *PM*2.5, and *SO*_2_ are included in the model in a linear relationship according to previous studies (16, 22, 33–35). Mean relative humidity (RH) was used as a natural cubic spline function as a connection with 3 degrees of freedom (9). With reference to previous studies, The median daily temperature was used as a reference value to calculate the RR for different lag days of exposure to the four division point temperatures (14, 36).

We analyzed the effect of ambient temperature and specific environmental factors on stroke emergency department visits separately by GAM. In addition, subgroup analyses based on a multi-factor model explored the differences between ambient temperature and stroke visits across gender ( male and female), age (<65 and ≥65 years), and stroke subtype populations (IS and HS). To explore the robustness of the multi-factor model, we performed sensitivity analyses by setting the reference temperature to the ambient temperature that provided the lowest risk, as well as varying the degrees of freedom of the exposure dimension from 4 to 8 and the degrees of freedom of the lag dimension from 2 to 6.

The above data analysis and models were done in the “Hmisc,” ”dlnm,“ and ”mgcv“ packages of R software (version 4.1.2). All statistical tests were two-sided, and *p* < 0.05 was considered statistically significant.

## Results

### Descriptive analysis

Between 2017 and 2018, a total of 26,984 stroke patients were visited, with 17,388 IS patients and 9,596 HS patients. Table 1 shows the number of daily visits for stroke and subgroups, as well as the status of each daily environmental variable during the study period. Non-parametric tests between subgroups showed that the number of stroke visits was statistically significant between each subgroup.

**Table 1:** Summary statistics of the number of daily visits and description of environmental factors for stroke and subgroups in Beijing, 2017–2018.

	**Mean ± SD**	**Minimum**	***P*** **_25_**	**Median**	***P*** **_75_**	**Maximum**
Daily visits
Stroke	36.96 ± 9.19	13.00	30.00	37.00	43.00	75.00
IS	23.82 ± 6.95	7.00	19.00	24.00	28.00	50.00
HS	13.15 ± 4.49	2.00	10.00	13.00	16.00	30.00
Age<65	12.96 ± 4.30	3.00	10.00	13.00	16.00	29.00
Age≥65	24.00 ± 6.97	7.00	19.00	24.00	29.00	55.00
Male	21.92 ± 6.20	5.00	18.00	22.00	26.00	42.00
Female	15.04 ± 4.87	3.00	12.00	15.00	18.00	34.00
Meteorological factors
Mean temperature (°C)	12.07 ± 11.81	−12.00	0.48	13.72	22.97	30.90
TV (°C)	11.87 ± 4.09	2.23	8.70	11.65	14.92	23.80
Air pressure (hPa)	993.76 ± 9.45	974.10	985.50	994.50	1000.83	1019.10
Precipitation (mm)	1.61 ± 6.25	0.00	0.00	0.00	0.00	60.33
Wind speed (m/s)	1.70 ± 0.63	0.47	1.27	1.57	2.00	5.03
Relative humidity (%)	52.76 ± 19.47	14.67	36.33	52.76	70.00	94.67
Air pollution factors
$NO_{2}\;\left(\mu g/m^{3}\right)$	40.63 ± 19.58	6.00	27.00	36.00	50.00	145.00
$SO_{2}\;\left(\mu g/m^{3}\right)$	5.95 ± 6.45	1.00	2.00	4.00	7.00	81.00
$O_{2}\;\left(\mu g/m^{3}\right)$	61.60 ± 37.77	3.00	33.25	55.00	84.00	181.00
*CO* (μ*g*/*m*^3^)	0.87 ± 0.63	0.20	0.50	0.76	1.02	7.28
$PM_{2.5}\;\left(\mu g/m^{3}\right)$	52.58 ± 49.00	3.00	20.00	40.00	67.75	430.00
$PM_{10}\;\left(\mu g/m^{3}\right)$	80.92 ± 68.70	0.00	41.00	65.50	99.75	858.00

SD, standard deviation; *P*_*x*_, xth percentile; IS, ischemic stroke; HS, hemorrhagic stroke; TV, temperature variation.

**Figure 1:**
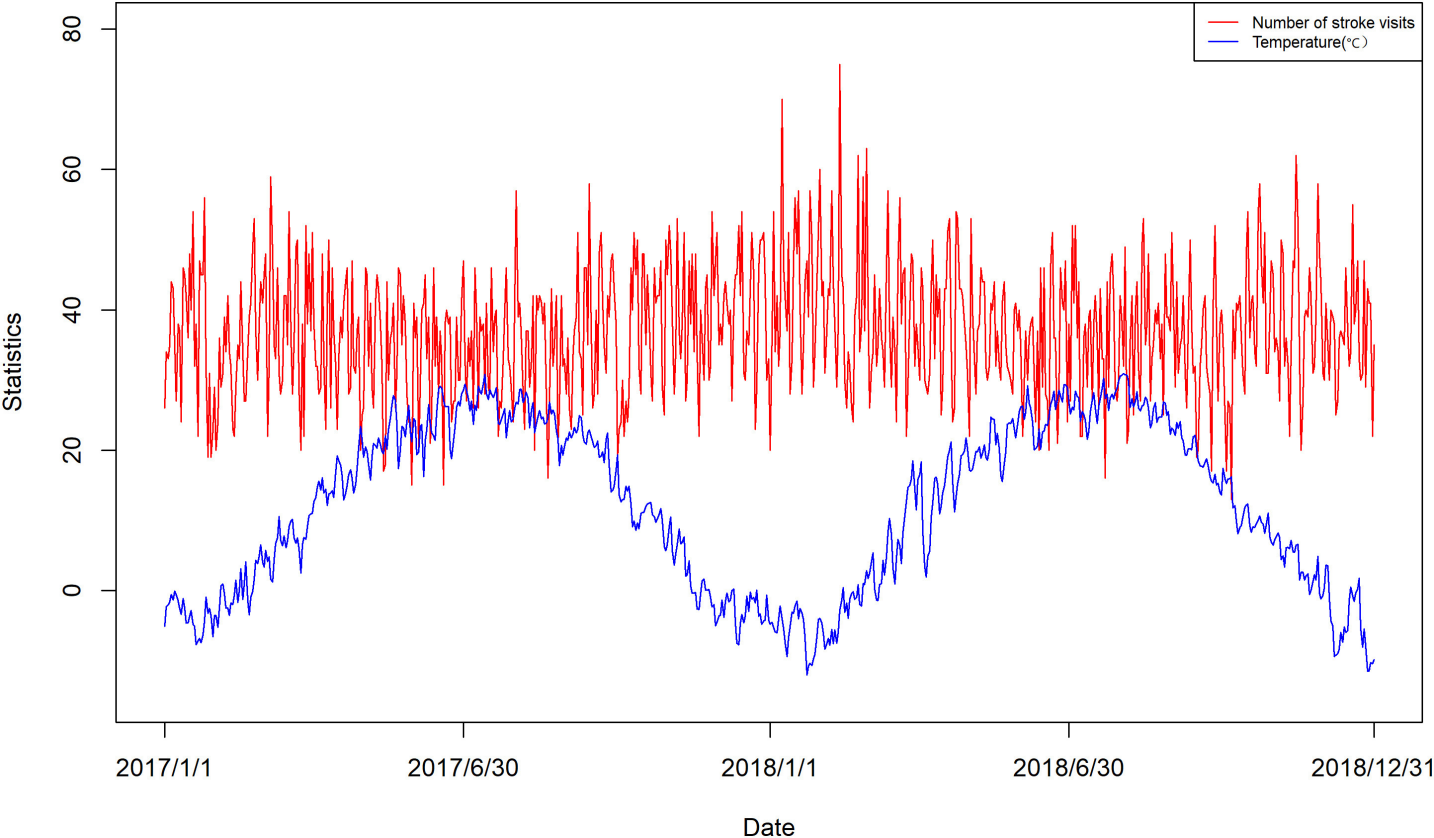
Time series distribution of daily number of stroke visits and mean daily temperature in Beijing.

Time series plot and bubble plot of stroke visits and daily mean temperature are displayed in Figure 1 and Supplementary Figure 1 respectively. The plots show that stroke visits follow a cyclical and seasonal pattern, with a trend toward greater numbers in the fall and winter, a negative correlation between stroke visits and ambient temperature, and relatively more visits under extreme ambient temperature exposure.

The GAM results demonstrate a non-linear relationship between meteorological factors such as mean temperature and mean relative humidity and the number of visits, and an approximately linear relationship between TV and most air pollution factors and the number of visits (Supplementary Figures 4, 5).

### Single DLNM

Median temperature is used as the reference temperature, the risk of stroke increased with either lower or higher temperatures (Supplementary Figure 2). Supplementary Figure 3 shows the lagged effect of dividing the point temperature based on the reference temperature, the RR for extreme cold (−10°C) and moderate cold (−4°C) reached the maximum on lag 14 with 1.20 (95% CI:1.09, 1.32) and 1.20 (95% CI:1.10, 1.30) respectively, the RR for extreme hot (30°C) and moderate hot (27°C ) both reached the maximum on lag 6, at 1.07 (95% CI: 1.04, 1.11) and 1.07 (95% CI: 1.03, 1.11), respectively.

Table 2 shows the cumulative effect of specific temperature on stroke incidence compared with the reference temperature. The RR of cold temperature gradually increased during the cumulative lag 0–14 days, and the RR of hot temperature peaked and then decreased after the cumulative lag 0–10 days. Specifically, extreme cold and moderate cold were 2.09 (95% CI: 1.16, 3.77) and 2.25 (95% CI: 1.33, 3.80) at lag 0–14 days, respectively, and the RR for moderate hot reached a maximum of 1.40 (95% CI: 1.06, 1.84) at lag 0–10 days, but the RR for extreme hot was not statistically significant.

**Table 2:** Cumulative effects of specific temperatures on stroke incidence in single DLNM at a specific lag structure.

	**Extreme cold** **P_1_**:** −10°C**	**Moderate cold** **P_10_**:**−4°C**	**Moderate hot** **P_90_: 27°C**	**Extreme hot** **P_99_**:** 30°C**
Lag 0–3	0.95 (0.75, 1.20)	0.93 (0.76, 1.14)	1.01 (0.87, 1.18)	0.97 (0.81, 1.15)
Lag 0–7	1.35 (0.95, 1.91)	1.27 (0.93, 1.74)	1.33* **(1.06, 1.67)**	1.24 (0.97, 1.58)
Lag 0–10	1.39 (0.89, 2.19)	1.42 (0.94, 2.13)	1.40* **(1.06, 1.84)**	1.33 (1.00, 1.77)
Lag 0–14	2.09* **(1.16, 3.77)**	2.25* **(1.33, 3.80)**	1.17 (0.82, 1.66)	1.12 (0.78, 1.62)

*p < 0.05 was considered statistically significant and is indicated in bold font.

### Multi-factor DLNM

The correlation is stronger when the correlation coefficient is >0.6. Strong correlations exist between air pressure and *O*_3_ and ambient temperature. In addition, the correlations between relative humidity and wind speed, *PM*_2.5_ and *PM*_10_, and *SO*_2_ and *CO* are strong, and covariance occurs if factors with strong correlations are included in the model at the same time (Supplementary Table 1). In this study, we finally combined the GAM results (Supplementary Figures 4, 5) and chose to select the daytime temperature variation, daily average relative humidity, *PM*_2.5_, and *SO*_2_ to be included in the multi-factor model.

**Figure 2:**
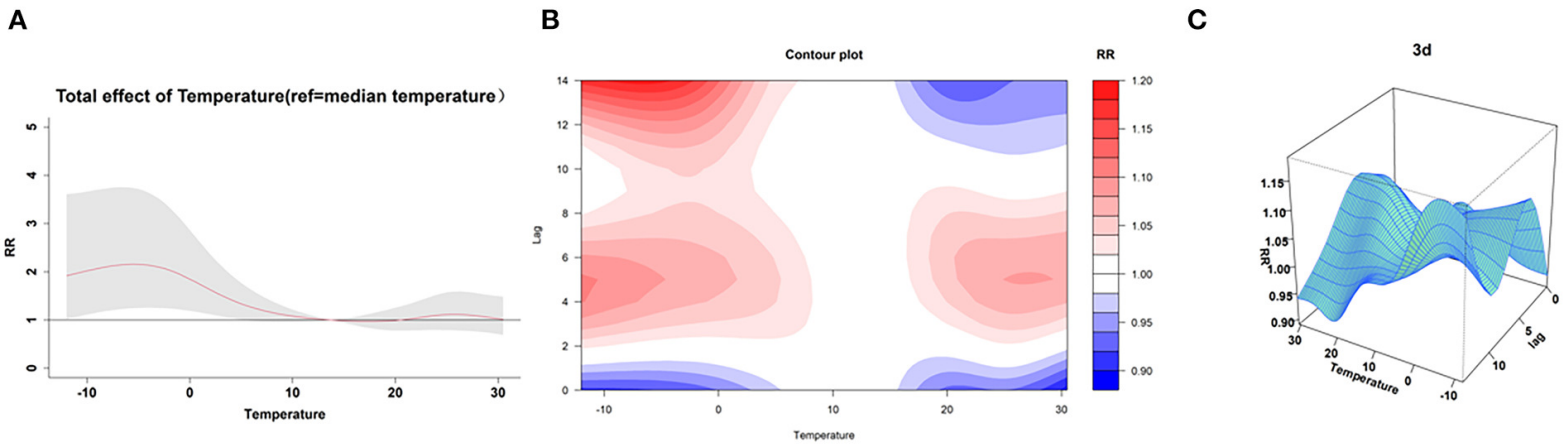
The visualization of the lag effect. **(A)** Shows that cold temperature significantly increased the risk of stroke compared to the median temperature. The contour plot **(B)** and 3D plot **(C)** of the lag effect show that the lag effect of cold temperature is strongest on day 14, while the lag effect of hot temperature is strongest on day 5 and day 6.

**Figure 3:**
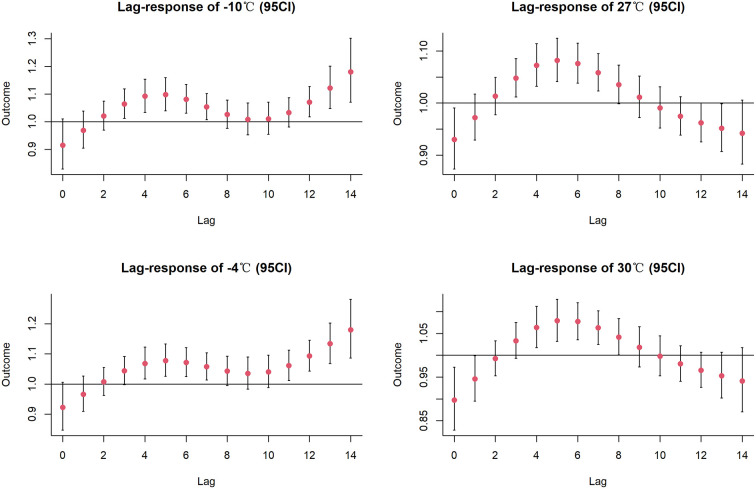
The visualization of the lagged effect at specific temperatures.

Figure 2 depicts the combined exposure-lag-response effect of mean temperature on the incidence of stroke in the multi-factor model. Compared to the reference temperature, the risk of stroke occurring increases for both cold and hot temperatures. Overall, compared to hot temperatures, cold temperatures had a higher impact and longer lag time. In the multi-factor model, the extreme cold and moderate cold effects were also largest at lag day 14 with RRs of 1.18 (95% CI: 1.07, 1.30) and 1.18 (95% CI: 1.09, 1.28), respectively; the extreme hot and moderate hot had the largest RRs at lag day 6 with RRs of 1.08 (95% CI: 1.04, 1.13) and 1.08 (95% CI: 1.03, 1.13), respectively (Figure 3).

The cumulative effect of cold temperature reached the maximum at lag 0–14 days, with 2.02 (95% CI: 1.11, 3.67) for extreme cold and 2.14 (95% CI: 1.25, 3.64) for moderate cold (Supplementary Table 2). The extreme hot and moderate hot reached the maximum at lag 0–10 days but were not statistically significant (Supplementary Table 2). Compared to the single model, the cumulative effects of specific temperatures at the same lag days were all decreased when controlling for the effects of other environmental factors. We then analyzed the interaction effect of temperature with other specific environmental factors in multi-factor DLNM on stroke visits by GAM (Supplementary Figure 6). The correction effect showed that at a certain ambient temperature, higher TV, higher *PM*_2.5_ concentration and lower *SO*_2_ concentration were linked to a higher risk of stroke incidence, while relative humidity showed a obviously negative association with the risk of stroke incidence within a certain interval.

### Subgroup analysis

According to Table 3, the cumulative effects of cold temperature in different subgroups all peaked at lags of 0–14 days, and the cumulative effects of hot temperature all peaked at lags of 0–10 days. The risk of stroke was greater in IS than in HS at low temperatures, and the effect of hot temperatures on the onset of HS was greater. Both low and high temperatures were more likely to affect those aged ≥ 65 years than those aged <65 years. Male exposed to high and moderate levels of cold temperature had a higher risk of stroke, with moderate cold exposure having the highest impact on the male at lag 0–14 days, 2.38 (95% CI: 1.19, 4.76). Stroke incidence in female was more affected by extreme cold at lag 0–14 days, 2.11 (95% CI: 0.84, 5.35), but not statistically significant.

**Table 3:** Cumulative effects of different subgroups on stroke incidence at specific ambient temperatures.

		**Extreme cold** **P_1_**:** −10°C**	**Moderate cold** **P_10_**:** −4°C**	**Moderate hot** **P_90_: 27°C**	**Extreme hot** **P_99_**:** 30°C**
Stroke type					
IS	Lag 0–3	1.06 (0.78, 1.42)	0.98 (0.75, 1.28)	0.96 (0.79, 1.18)	0.83 (0.65, 1.04)
	Lag 0–7	1.48 (0.95, 2.30)	1.31 (0.88, 1.96)	1.25 (0.94, 1.68)	1.08 (0.79, 1.48)
	Lag 0–10	1.60 (0.90, 2.83)	1.56 (0.93, 2.62)	1.31 (0.92, 1.86)	1.15 (0.79, 1.66)
	Lag 0–14	2.33* **(1.10,4.92)**	2.36* **(1.20, 4.62)**	1.15 (0.74, 1.80)	0.98 (0.62, 1.56)
HS	Lag 0–3	0.82 (0.55, 1.21)	0.87 (0.61, 1.23)	0.95 (0.73, 1.24)	0.97 (0.71, 1.33)
	Lag 0–7	1.07 (0.60, 1.92)	1.09 (0.65, 1.84)	1.29 (0.88, 1.90)	1.28 (0.84, 1.95)
	Lag 0–10	1.06 (0.50, 2.26)	1.11 (0.56, 2.18)	1.32 (0.83, 2.11)	1.35 (0.82, 2.23)
	Lag 0–14	1.56 (0.58, 4.19)	1.80 (0.75, 4.31)	1.02 (0.56, 1.85)	1.15 (0.61, 2.16)
Age					
<65	Lag 0–3	0.89 (0.59, 1.33)	0.97 (0.68, 1.39)	0.88 (0.68, 1.15)	0.80 (0.59, 1.10)
	Lag 0–7	1.12 (0.61, 2.03)	1.31 (0.77, 2.25)	1.23 (0.83, 1.81)	1.05 (0.69, 1,59)
	Lag 0–10	1.02 (0.47, 2.21)	1.26 (0.63, 2.53)	1.26 (0.79, 2.02)	1.04 (0.63, 1.70)
	Lag 0–14	1.44 (0.52, 3.99)	1.99 (0.81, 4.90)	1.16 (0.64, 2.10)	0.93 (0.50, 1.74)
≥65	Lag 0–3	1.00 (0.75, 1.34)	0.92 (0.71, 1.19)	1.01 (0.83, 1.23)	0.91 (0.72, 1.15)
	Lag 0–7	1.43 (0.93, 2.20)	1.18 (0.80, 1.75)	1.30 (0.97, 1.74)	1.20 (0.88, 1.65)
	Lag 0–10	1.61 (0.92, 2.83)	1.45 (0.87, 2.41)	1.36 (0.95, 1.93)	1.32 (0.91, 1.91)
	Lag 0–14	2.39* **(1.15, 5.00)**	2.22* **(1.15, 4.31)**	1.09 (0.70, 1.70)	1.09 (0.68, 1.74)
Gender				
Male	Lag 0–3	0.87 (0.64, 1.19)	0.91 (0.69, 1.20)	0.97 (0.79, 1.19)	0.83 (0.65, 1.06)
	Lag 0–7	1.23 (0.78, 1.95)	1.22 (0.80, 1.84)	1.35* **(1.00, 1.82)**	1.14 (0.82, 1.58)
	Lag 0–10	1.30 (0.72, 2.36)	1.51 (0.89, 2.59)	1.52* **(1.06, 1.93)**	1.26 (0.86, 1.85)
	Lag 0–14	1.94 (0.90, 4.21)	2.38* **(1.19, 4.76)**	1.49 (0.93, 2.19)	1.23 (0.76, 2.03)
Female	Lag 0–3	1.11 (0.77, 1.60)	0.97 (0.70, 1.35)	0.94 (0.73, 1.21)	0.93 (0.70, 1.25)
	Lag 0–7	1.44 (0.83, 2.48)	1.23 (0.75, 2.01)	1.17 (0.81, 1.68)	1.16 (0.78, 1.72)
	Lag 0–10	1.47 (0.72, 3.00)	1.19 (0.62, 2.26)	1.08 (0.69, 1.67)	1.15 (0.72, 1.83)
	Lag 0–14	2.11 (0.84, 5.35)	1.80 (0.78, 4.14)	0.73 (0.42, 1.27)	0.80 (0.44, 1.43)

*p < 0.05 was considered statistically significant and is indicated in bold font.

IS, ischemic stroke; HS, hemorrhagic stroke.

### Sensitivity analysis

The temperature that provided the lowest risk of stroke visits in this study was 17°C. By re-analyzing in the multi-factor DLNM with 17°C as the reference temperature, the results showed the cumulative-exposure-lag effect of temperature on stroke visits was not found to be significantly altered (See Supplementary Tables 3, 4). The sensitivity analysis is also by changing the degree of freedom of the natural cubic spline function of the lag dimension and the exposure dimension of the cross basis in the DLNM. In the previous analysis, the values were 4 and 6 respectively, which followed the AIC minimization (Supplementary Figure 7). The effect values did not change significantly when we took the lag dimension degrees of freedom from 2 to 6 and the exposure dimension from 4 to 8 (Supplementary Figure 8). These analyses all indicated that the parameters of the DLNM model developed in our study were chosen reasonably and the model fitting results were more robust.

## Discussion

As far as we know, this is the first study to use DLNM to explore how daily mean temperature affects emergency stroke visits in Beijing. After a two-stage analysis, we confirmed the direct and lagged effects of cold and hot temperatures on the risk of stroke incidence, and the effect of increased cold temperatures was more significant, but after correction for other environmental factors, we only observed a cumulative effect of cold temperatures on stroke incidence.

Extensive epidemiological evidence suggests that cold and hot exposures show a significant association with the risk of stroke mortality, and a study that combined the acute effects of ambient temperature on stroke mortality in 12 counties in Hubei Province, China, revealed a general inverse J-shaped connection between cold and heat waves and an increase in stroke mortality, with findings that were consistent with those of other research (5, 37–39). The association between ambient temperature and morbidity risk has received less attention in research, yet morbidity risk can reflect more direct productivity loss, patient and healthcare costs than mortality risk. In previous studies from China, a Taiwan study reported that the risk of hospitalization for HS on a cold day was approximately twice that of a warm day (40), and a study from Hong Kong found that a 1°C decrease in mean temperature was associated with a 2.7% increase in stroke admissions in the range of 8.2–31.8°C (41). A study analyzed stroke patients from 67 sentinel hospitals in Guangzhou from 2013–2015 and found that the attributable risk of IS and HS caused by cold temperature were 9.06% and 15.09%, respectively (9). In consistent with the findings above, our data revealed a statistically significant positive correlation between cold temperature and the incidence of stroke. The mechanism of the effect of cold temperature may be due to the fact that cold temperature tends to induce cerebral vasoconstriction, blood pressure fluctuations, and elevated circulatory levels, which may contribute to the occurrence of stroke (9).

After correction for multiple environmental factors in phase 2, both the direct and lagged effects of cold and hot temperatures on stroke risk were attenuated, and the effect of hot temperatures became non-significant, our results broadly agree with those of the one earlier study conducted in Beijing (14), which found that extreme cold temperature (P_1_: −9.6°C) compared with 1.2°C (P_25_) increased the cumulative RR for IS at lag 0–14 days, The cumulative RR was 0.51 (95 % CI: 0.08–1.10) higher for IS and 0.28 (95 % CI: 0.03–0.59) higher for HS at lag 0–3 days compared with 1.2°C (P_25_), similarly, this study did not find an effect of hot on stroke incidence. In contrast, our study found a greater RR of cold temperature on IS incidence at lag 0–14 days, and in addition we did not find a significant effect of ambient temperature on HS. Some of the reasons why our study differs from it may be the following, firstly, the lag structure and reference temperature settings of the two studies were different, which led to the difference in the final RR values, secondly, the factors included in the multi-factor model were different, we took into account the effects of TV and *SO*_2_ in addition to relative humidity and *PM*_2.5_ , and the subsequent analysis showed that these factors did interact with ambient temperature. Our study used emergency data rather than hospitalization data, as stroke is an acute and serious condition with rapid incidence and progression, so the use of emergency visits can more accurately and reasonably reflect the risk of stroke due to the corrective effect of environmental factors. According to domestic and international studies related to the effect of ambient temperature on stroke emergency department visits, a study from Japan found that each 1°C drop in ambient temperature increased IS and HS visits by 3.56% and 35.57%, respectively (42), and another study from Turkey also found that lower ambient temperatures increased stroke visits (43). Additionally, a research from Brisbane, Australia, revealed no statistically significant link between high temperatures and admissions for emergency stroke (44), and the results of these studies, which were based on emergency data, were the same as our results.

The lag effect analysis results showed that neither the single-factor model nor the multi-factor model changed the lag days of the maximum effect, with the cold temperature effect being maximum at lag 14 and lag 0–14 days and the hot temperature effect being maximum at lag 6 and lag 0–10 days, indicating that the cold temperature effect persisted over a longer lag period while the hot temperature effect was relatively weak and acted within a shorter period. Similarly, a previous study from Beijing also found that hot temperature effects behave acutely relative to cold temperatures (14). Health care resource allocation and the development of public health interventions can be informed by this lag structure. Preventive measures in the short term can help address the health risk of stroke incidence under the effects of hot temperatures, while long-term protection can target the risk of stroke incidence under the effects of cold temperatures. In addition, this can be used to estimate the cost of health service utilization. Based on our data, we conclude that the relative risk of extreme cold exposure in Beijing in 2017–2018 is lower than that of moderate cold temperatures in emergency department visits for stroke, and we speculate that this finding may be due to the effect of early warning. Public health authorities have previously implemented preventive and protective measures mainly for extreme weather (45), so people are more sensitive to extreme cold temperatures and are more likely to take protective measures, such as going out less, actively adding clothing, and increasing heating, which implies that health problems caused by moderate cold temperatures should receive more attention in the future and public health authorities should further develop defensive measures against moderate cold temperatures.

In the second phase of the analysis, with the analysis in the GAM, we also focused on how ambient temperature and other environmental factors affect the development of stroke. We found that higher TV increases the risk of stroke, following the findings from Bangladesh, which found a 1.00% increase in emergency department visits for cardiovascular disease per 1°C increase in TV (17), with possible underlying mechanisms being unstable changes in ambient temperature can disrupt the body's thermoregulatory balance, thereby reducing the ability to adapt to ambient temperature, and in addition TV is associated with peripheral vasoconstriction, platelet viscosity, and the ability of the immune system to resist infectious agents (46). We observed a positive association between *PM*_2.5_ and the risk of stroke, which is also consistent with previous studies (18, 47). In our original data, the relative humidity in Beijing was distributed between 60% and 80% for many days, so for this interval, the lower the relative humidity the higher the risk of stroke, a finding that is consistent with previous studies (48, 49). However, we found that *SO*_2_ was negatively associated with stroke incidence, which is contrary to the previous positive findings (47, 50), probably because these studies were on the effect of *SO*_2_ on IS, but we did not investigate the effect of *SO*_2_ on stroke subtypes, perhaps because of the confounding effect between subtypes. In addition, it has been reported that there is no significant relationship between *SO*_2_ and stroke visits (51, 52), so the relationship between *SO*_2_ and stroke or stroke subtypes is not conclusive, and further studies are needed to provide a reasonable and accurate basis.

Subgroup analysis revealed a positive correlation between the effects of low temperature on different subtypes, and no change in the lagged pattern of cold and hot effects, which is consistent with previous findings (9, 14, 40, 41). This is different from previous studies from Taiwan and Hong Kong (40, 41), our study found that the effect of cold temperature on the incidence of IS was greater than that of HS, possibly because both studies were from subtropical monsoon climate regions, whereas Beijing is a typical temperate monsoon climate, and there is a large difference between the mean temperatures of cold and hot months in both, with temperatures generally higher in subtropical monsoon climate regions than in Beijing, it is possible that the association between cold temperatures below a certain threshold on stroke subtypes was not observed. It is also possible that factors such as urban characteristics, air pollution levels, and heating facilities in the north and south may be relevant, but it is certain that there are differences in the effects of ambient temperature on stroke subtypes among each other. The analysis of subgroups of people of different ages and genders showed that the effect of cold temperatures was more significant in the elderly and male population, with an RR of 2.39 and 2.38, respectively, which is in full agreement with the results of a previous study from Guangzhou (9), possible reasons for this result are decreased metabolic levels, reduced thermoregulatory capacity, or co-morbidities in the elderly; the difference between the genders may be due to different hormone levels in males and females, and the fact that males are generally more likely to work outdoors and have more exposure to ambient temperature or other environmental factors (53).

Our study has obvious advantages. First, we applied an advanced statistical method, DLNM can more accurately and comprehensively reflect the effect of ambient temperature on stroke incidence, including quantitative assessment of the lagged effect and the cumulative effect of ambient temperature. Finally, because our data are newer, large-scale emergency department visits, they are a more accurate representation of stroke incidence than inpatient data, and to our knowledge, we are the only study to explore the effect of temperature on stroke emergency department visits in Beijing, which may provide a useful tool for public health research in Beijing. This may not only offer the department of public health a scientific foundation for developing relevant policies, but also provide new ideas for improving hospital emergency room prediction models with temperature as a predictor. Of course, our study has some limitations, as other similar studies are ecological studies and cannot avoid ecological fallacies; there are differences between the exposure levels of the population and the actual exposure of individuals; secondly, due to different geographical characteristics, our findings need to be extrapolated to areas outside Beijing with caution; and finally, because stroke incidence is influenced by socioeconomic conditions, literacy, and other co-morbidities in addition to environmental factors, however, our study did not collect and analyze these possible confounding factors.

## Conclusions

In summary, our study shows that there is a significant non-linear relationship between temperature and stroke incidence, with cold temperatures having a stronger and longer-lasting effect on stroke incidence compared with hot temperatures. In addition, larger TV, higher *PM*_2.5_, and lower relative humidity increased the risk of stroke occurrence. These findings have important public health and clinical implications for the development of stroke control strategies in Beijing under different weather conditions.

## Data availability statement

The data that support the findings of this study are available from the corresponding author, upon reasonable request.

## Ethics statement

This study was approved by the Ethics Committees of Tianjin Medical University (No. TMUhMEC 2021009). Overall aggregated data were used in our study and no information about individual patient privacy was involved in the analysis.

## Author contributions

JZ, ZC, and WZ: conceptualization, data curation, investigation, and writing-original draft. YZ: conceptualization, investigation, and writing-original draft. YN: validation and formal analysis. JH, JW, and XL: writing-review & editing. CL and YG: conceptualization and writing-review & editing. ZC and WZ: writing-review & editing and supervision. All authors contributed to the article and approved the submitted version.

## Conflict of interest

The authors declare that the research was conducted in the absence of any commercial or financial relationships that could be construed as a potential conflict of interest.

## Publisher's note

All claims expressed in this article are solely those of the authors and do not necessarily represent those of their affiliated organizations, or those of the publisher, the editors and the reviewers. Any product that may be evaluated in this article, or claim that may be made by its manufacturer, is not guaranteed or endorsed by the publisher.

## Abbreviations

GAM: Generalized additive model
DLNM: Distributed lagged non-linear model
CI: Confidence Interval
RR: Relative risk
IS: Ischemic stroke
HS: Hemorrhagic stroke
AIC: Akaike information criterion
TV: Temperature variation.
