# Supplementary material for: Effect of ambient temperature and other environmental factors on stroke emergency department visits in Beijing: A distributed lag non-linear model
Source: Front Public Health. 2022 Nov 16;10:1034534. doi: 10.3389/fpubh.2022.1034534 (PMC9709270; doi:10.3389/fpubh.2022.1034534)
Supplement: Supplementary file 1 [file Data_Sheet_1.pdf]

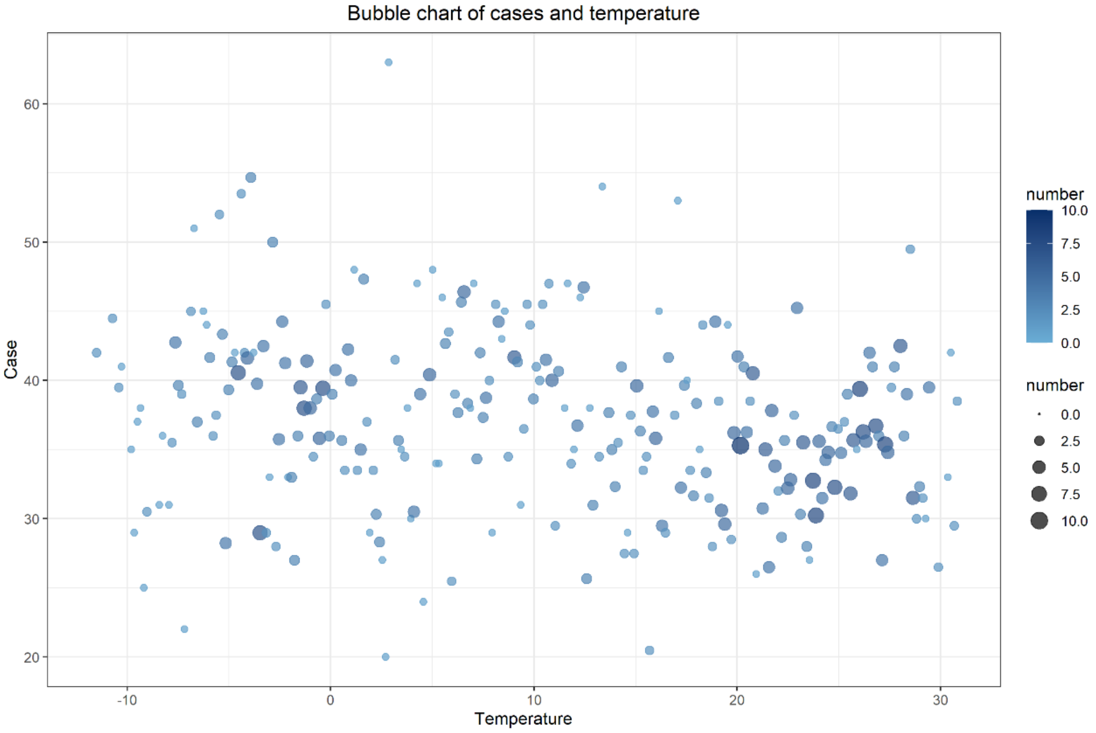

Figure S1 Bubble plot of the daily number of stroke visits versus mean daily temperature

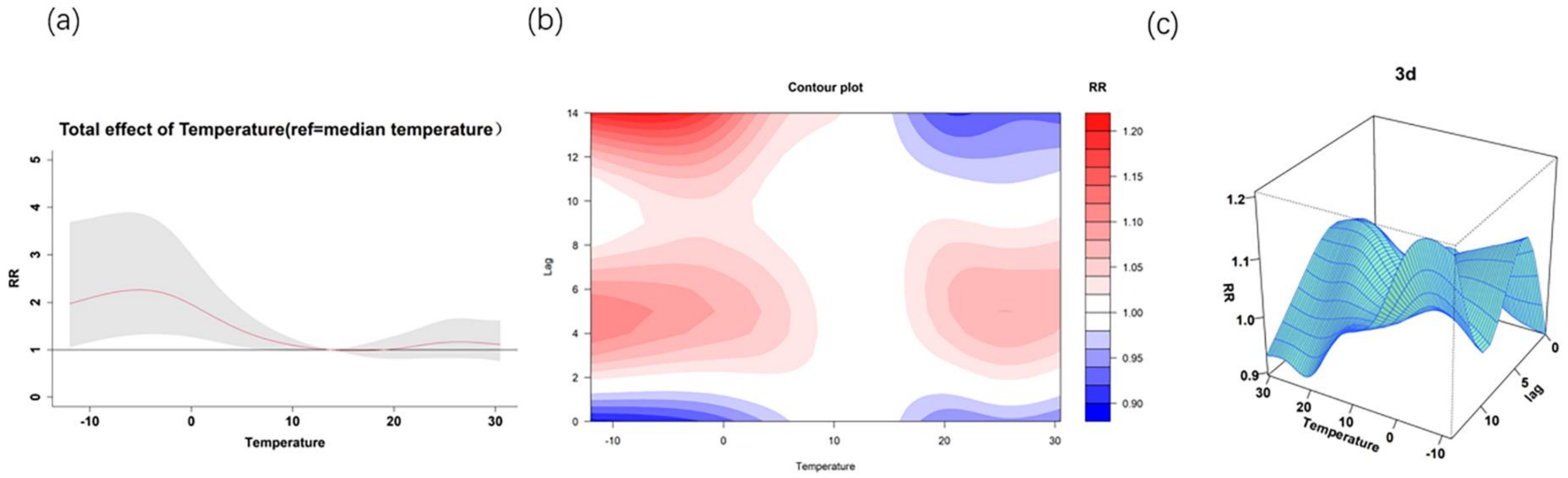

Figure S2 Joint exposure-lag-response plot of mean temperature on stroke incidence in single-factor model

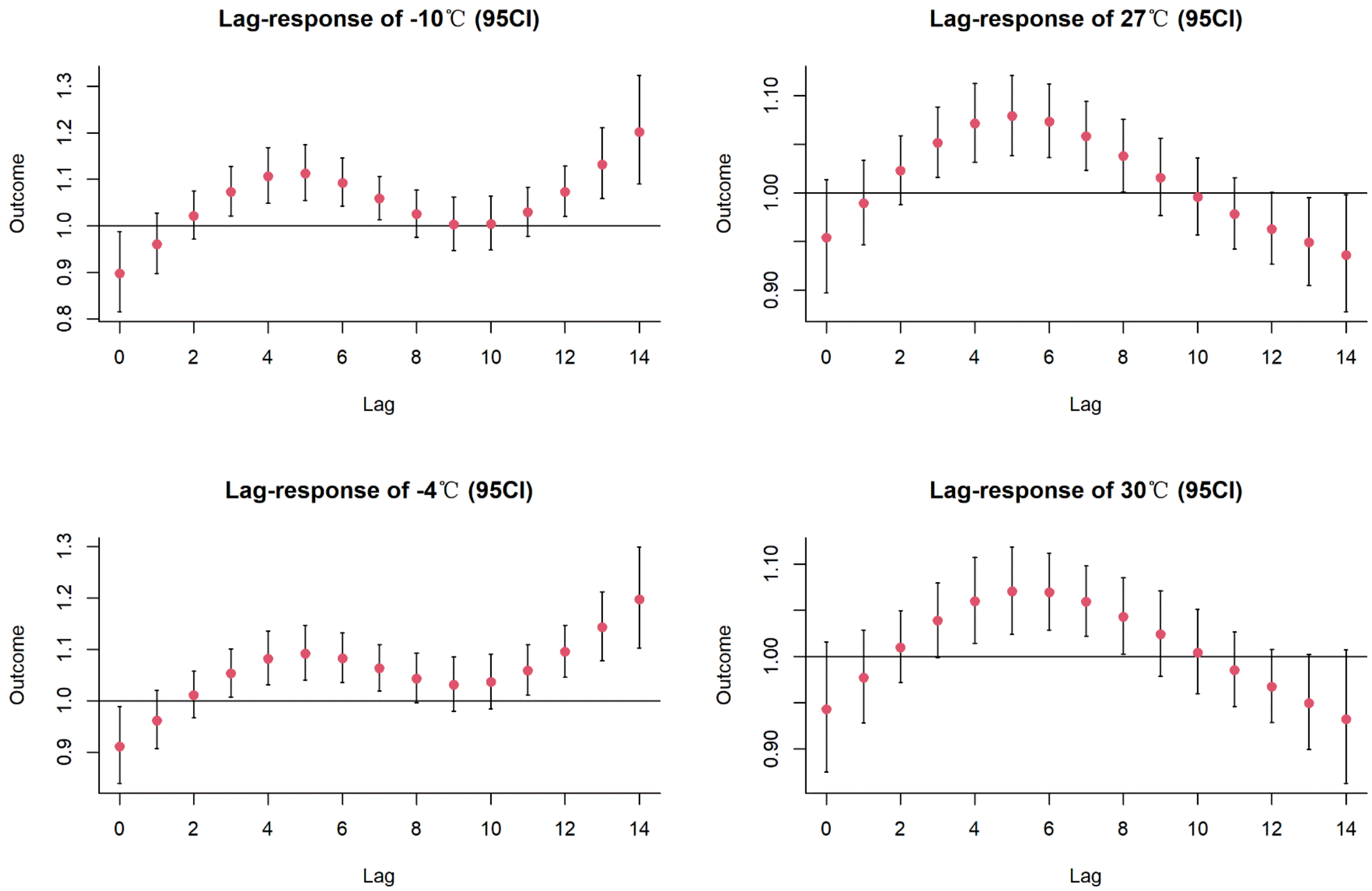

Figure S3 Plot of lagged effects of different lagged days for a specific temperature in the single-factor model

Table S1 Analysis of correlation between various environmental factors and with the number of stroke visits

	Visits of stroke	Temperature	Temperature variability	Relative humidity	Air pressure	Wind speed	Precipitation	NO2	O3	PM10	PM2.5	SO2	CO
Visits of stroke	1.00												
Temperature	**-0.14***	1.00											
Temperature variability	0.04	-0.07	1.00										
Relative humidity	-0.06	**0.50***	**-0.39***	1.00									
Air pressure	**0.11***	**-0.88***	-0.01	**-0.41***	1.00								
Wind speed	0.02	**-0.19***	0.03	**-0.65***	**0.12***	1.00							
Precipitation	**-0.16***	**0.38***	**-0.44***	**0.54***	**-0.36***	**-0.16***	1.00						
NO2	0.07	**-0.25***	**0.37***	**0.12***	**0.17***	**-0.44***	**-0.22***	1.00					
O3	**-0.12***	**0.74***	-0.01	0.08	**-0.70***	**0.20***	**0.21***	**-0.47***	1.00				
PM10	-0.04	0.06	**0.23***	**0.04***	**-0.16***	**-0.12***	**-0.15***	**0.61***	0.04	1.00			
PM2.5	-0.04	**0.09***	0.02	**0.40***	**-0.17***	**-0.37***	0.05	**0.65***	-0.03	**0.78***	1.00		
SO2	0.04	**-0.45***	**0.28***	**-0.27***	**0.31***	0.02	**-0.30***	**0.61***	**-0.29***	**0.52***	**0.46***	1.00	
CO	0.01	-0.05	-0.06	**0.45***	-0.02	**-0.48***	0.07	**0.69***	**-0.23***	**0.58***	**0.85***	**0.55***	1.00

$p<0.05$ was considered statistically significant and is indicated in bold font*

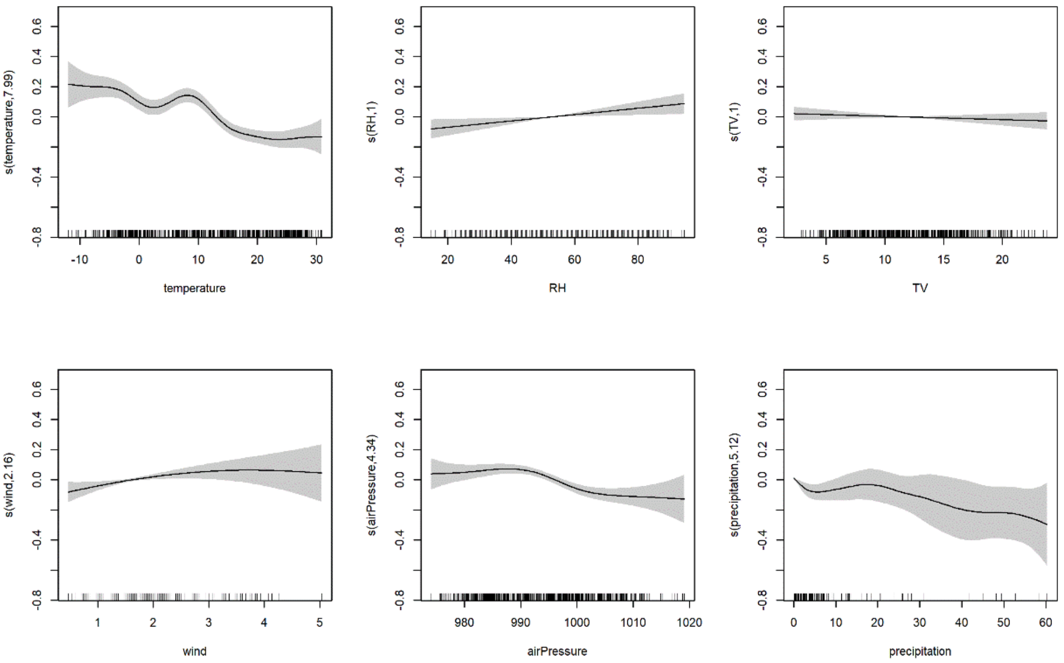

Figure S4 Plot of GAM results for each meteorological factor and number of stroke visits

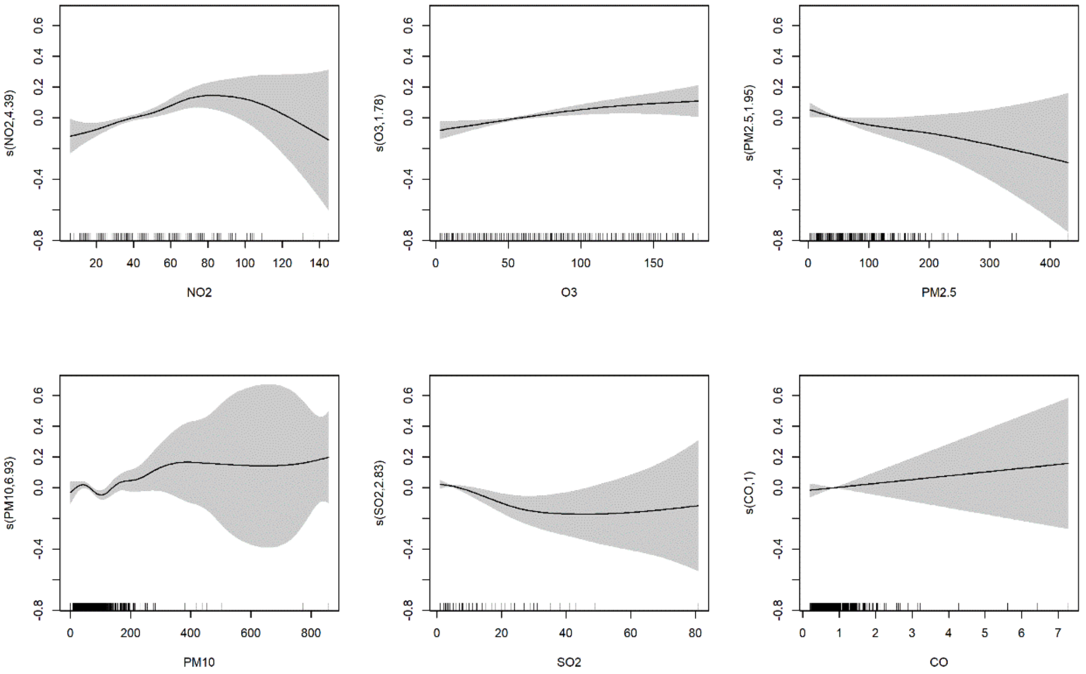

Figure S5 Plot of GAM results for each air pollution factor and number of stroke visits

Table S2 Cumulative effects of specific temperatures on stroke incidence in multiple-factor DLNM at a specific lag structure

	Extreme cold<br>P_1:-10℃	Moderate cold<br>P_{10}:-4℃	Moderate hot<br>P_{90}:27℃	Extreme hot<br>P_{99}:30℃
Lag0-3	0.96<br>(0.76, 1.22)	0.94<br>(0.76, 1.16)	0.96<br>(0.82, 1.13)	0.87<br>(0.72, 1.05)
Lag0-7	1.32<br>(0.93, 1.87)	1.23<br>(0.89, 1.68)	**1.27***<br>**(1.01, 1.60)**	1.14<br>(0.89, 1.47)
Lag0-10	1.38<br>(0.87, 2.18)	1.38<br>(0.91, 2.08)	1.32<br>(0.99, 1.74)	1.21<br>(0.90, 1.63)
Lag0-14	**2.02***<br>**(1.11, 3.67)**	**2.14***<br>**(1.25, 3.64)**	1.11<br>(0.77, 1.58)	1.03<br>(0.71, 1.49)

$p<0.05$ was considered statistically significant and is indicated in bold font*

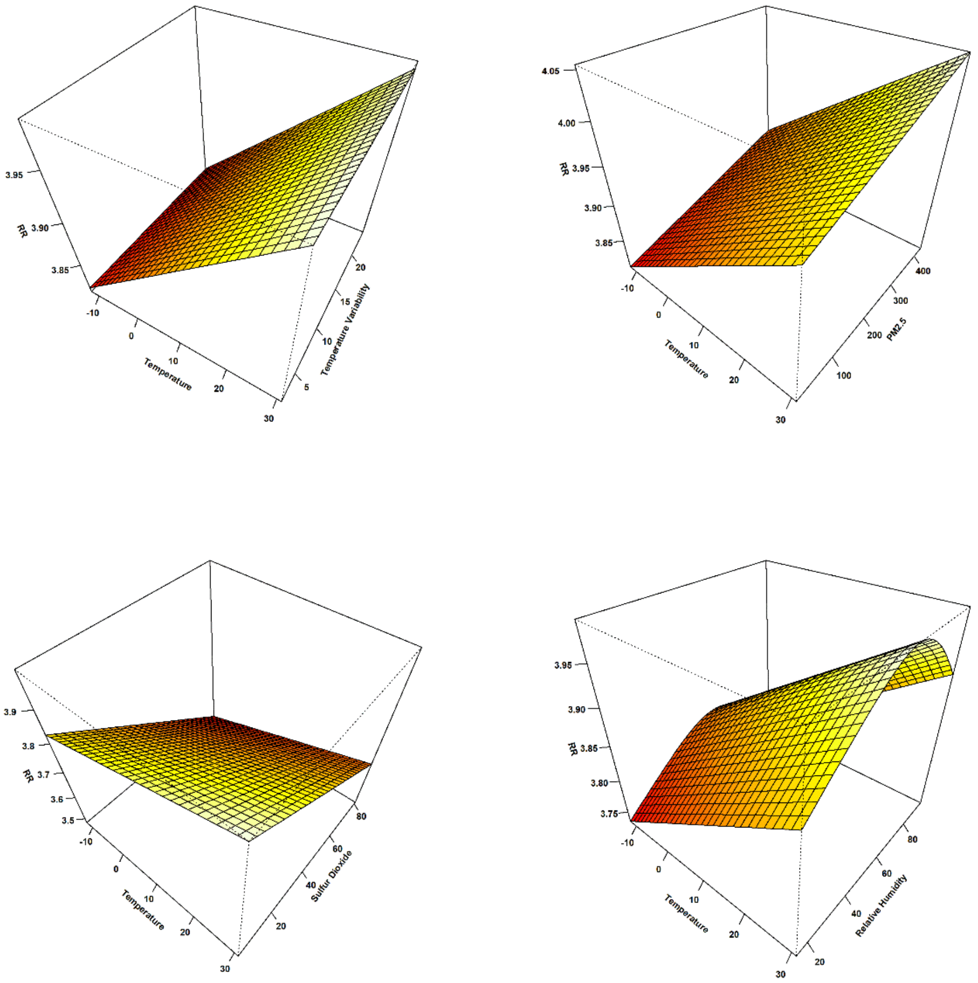

Figure S6 Effect of remaining environmental factors interacting with temperature on

stroke incidence

Table S3 Cumulative effects of specific temperatures on stroke incidence in the single DLNM (reference=17°C)

	Extreme cold<br>P_1:-10℃	Moderate cold<br>P_{10}:-4℃	Moderate hot<br>P_{90}:27℃	Extreme hot<br>P_{99}:30℃
Lag0-3	0.99<br>(0.78, 1.25)	0.98<br>(0.79, 1.20)	1.06<br>(0.94, 1.20)	1.01<br>(0.88, 1.17)
Lag0-7	1.28<br>(0.92, 1.80)	1.21<br>(0.89, 1.64)	**1.27*<br>(1.06, 1.52)**	1.18<br>(0.98, 1.43)
Lag0-10	1.28<br>(0.83, 1.97)	1.30<br>(0.88, 1.92)	**1.28*<br>(1.02, 1.60)**	1.22<br>(0.97, 1.54)
Lag0-14	**2.13*<br>(1.21, 3.78)**	**2.29*<br>(1.39, 3.79)**	1.19<br>(0.88, 1.60)	1.14<br>(0.84, 1.55)

p<0.05 was considered statistically significant and is indicated in bold font*

Table S4 Cumulative effects of specific temperatures on stroke incidence in the multi-factor DLNM (reference=17°C)

	Extreme cold<br>P_1:-10℃	Moderate cold<br>P_{10}:-4℃	Moderate hot<br>P_{90}:27℃	Extreme hot<br>P_{99}:30℃
Lag0-3	1.03<br>(0.81, 1.30)	1.00<br>(0.80, 1.24)	1.02<br>(0.90, 1.16)	0.93<br>(0.80, 1.08)
Lag0-7	1.28<br>(0.91, 1.81)	1.20<br>(0.88, 1.63)	**1.24*<br>(1.03, 1.49)**	1.11<br>(0.91, 1.36)
Lag0-10	1.29<br>(0.83, 2.00)	1.29<br>(0.87, 1.92)	1.23<br>(0.98, 1.55)	1.13<br>(0.89, 1.44)
Lag0-14	**2.09*<br>(1.17, 3.72)**	**2.21*<br>(1.33, 3.68)**	1.14<br>(0.85, 1.55)	1.06<br>(0.78, 1.45)

p<0.05 was considered statistically significant and is indicated in bold font*

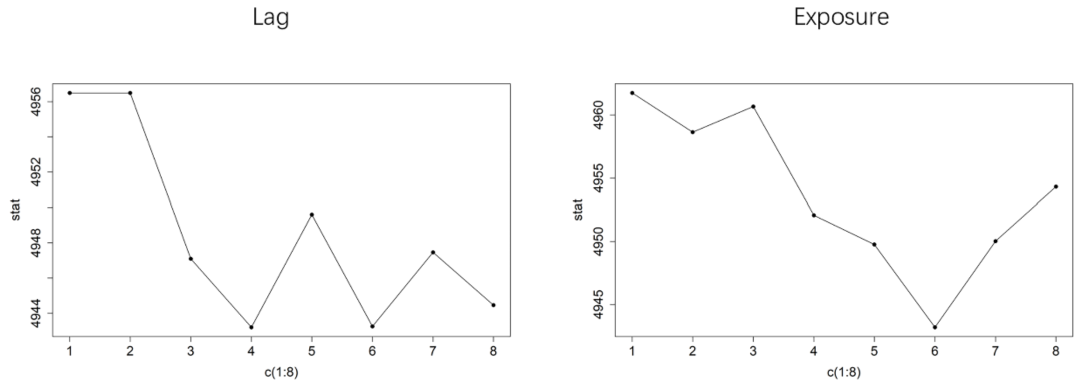

Figure S7 AIC values of the lag dimension and exposure dimension degrees of freedom in multiple-factor DLNM

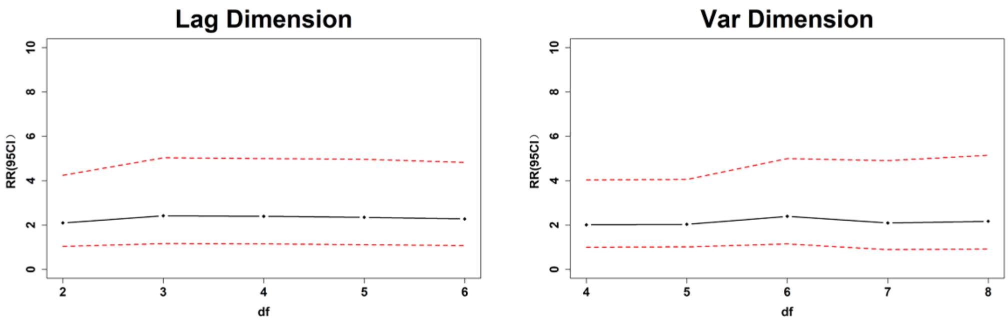

Figure S8 Sensitivity analysis of the effect of ambient temperature on stroke incidence at different relevant parameters in the DLNM
